# Supplementary material for: Clinical and genomic landscape of gastric cancer with a mesenchymal phenotype
Source: Nat Commun. 2018 May 3;9:1777. doi: 10.1038/s41467-018-04179-8 (PMC5934392; doi:10.1038/s41467-018-04179-8)
Supplement: Supplementary file 1 — Supplementary Information [file 41467_2018_4179_MOESM1_ESM.pdf]

Supplementary Information for

Clinical and genomic landscape of gastric cancer with a mesenchymal phenotype

Sang Cheul Oh[1,2,19], Bo Hwa Sohn[1,3,19], Jae-Ho Cheong[4,19], Sang-Bae Kim[1,3,19], Jae Eun Lee[4], Ki Cheong Park[4], Sang Ho Lee[5], Jong-Lyul Park[6], Yun-Yong Park[7], Hyun-Sung Lee[1,3], Hee-Jin Jang[1,3], Eun Sung Park[8], Sang-Cheol Kim[9], Jeonghoon Heo[10], In-Sun Chu[11], You-Jin Jang[12], Young-Jae Mok[12], WonKyung Jung[12], Baek-Hui Kim[13], Aeree Kim[13], Jae Yong Cho[14], Jae Yun Lim[14], Yuki Hayashi[15], Shumei Song[15], Elena Elimova[15], Jeannelyn S. Estralla[15], Jeffrey H. Lee[15], Manoop S. Bhutani[15], Yiling Lu[1,3], Wenbin Liu[1,3], Jeeyun Lee[17], Won Ki Kang[17], Sung Kim[18], Sung Hoon Noh[4], Gordon B. Mills[1,3], Seon-Young Kim[6], Jaffer A. Ajani[15], and Ju-Seog Lee[1,3].

[1]Department of Systems Biology, The University of Texas MD Anderson Cancer Center, Houston, Texas, 77030, USA.
[2]Division of Hemato-Oncology, Department of Internal Medicine, Guro Hospital, College of Medicine, Korea University, Seoul, 08308, Korea.
[3]Institute for Personalized Cancer Therapy, The University of Texas MD Anderson Cancer Center, Houston, Texas, 77030, USA.
[4]Department of Surgery, Yonsei University College of Medicine, Seoul, 03722, Korea
[5]Department of Surgery, Kosin University, College of Medicine, Busan, 49267, Korea
[6]Personalized Genomic Medicine Research Center, Korea Research Institute of Bioscience and Biotechnology, Daejeon, 34141, Korea
[7]ASAN Institute for Life Sciences, ASAN Medical Center, Department of Medicine, University of Ulsan College of Medicine, Seoul, 05505, Korea
[8]Medical research institute, college of medicine, Inha University, Incheon, 22212, Korea
[9]Department of Biomedical Informatics, Center for Genome Science, National Institute of Health, Daejeon, 34141, Korea
[10]Department of Molecular Biology and Immunology, Kosin University, College of Medicine, Busan, 49267, Korea
[11]Korean Bioinformation Center, Korea Research Institute of Bioscience and Biotechnology, Daejeon, 34141, Korea
[12]Department of Surgery, Guro Hospital, College of Medicine, Korea University, Seoul, 08308, Korea.
[13]Department of Pathology, Guro Hospital, College of Medicine, Korea University, Seoul, 08308, Korea.
[14]Medical Oncology, Yonsei University College of Medicine, Seoul, 03722, Korea
[15]Department of Gastrointestinal Medical Oncology, The University of Texas MD Anderson Cancer Center, Houston, Texas, 77030, USA.
[16]Department of Gastroenterology, Hepatology, and Nutrition, The University of Texas MD Anderson Cancer Center, Houston, Texas, 77030, USA.
[17]Division of Hematology-Oncology, Department of Medicine, Samsung Medical Center, Gangnam-Gu, Seoul, 06351, Korea.
[18]Department of Surgery, Samsung Medical Center, Gangnam-Gu, Seoul, 06351, Korea

[19]These authors contributed equally to this work.

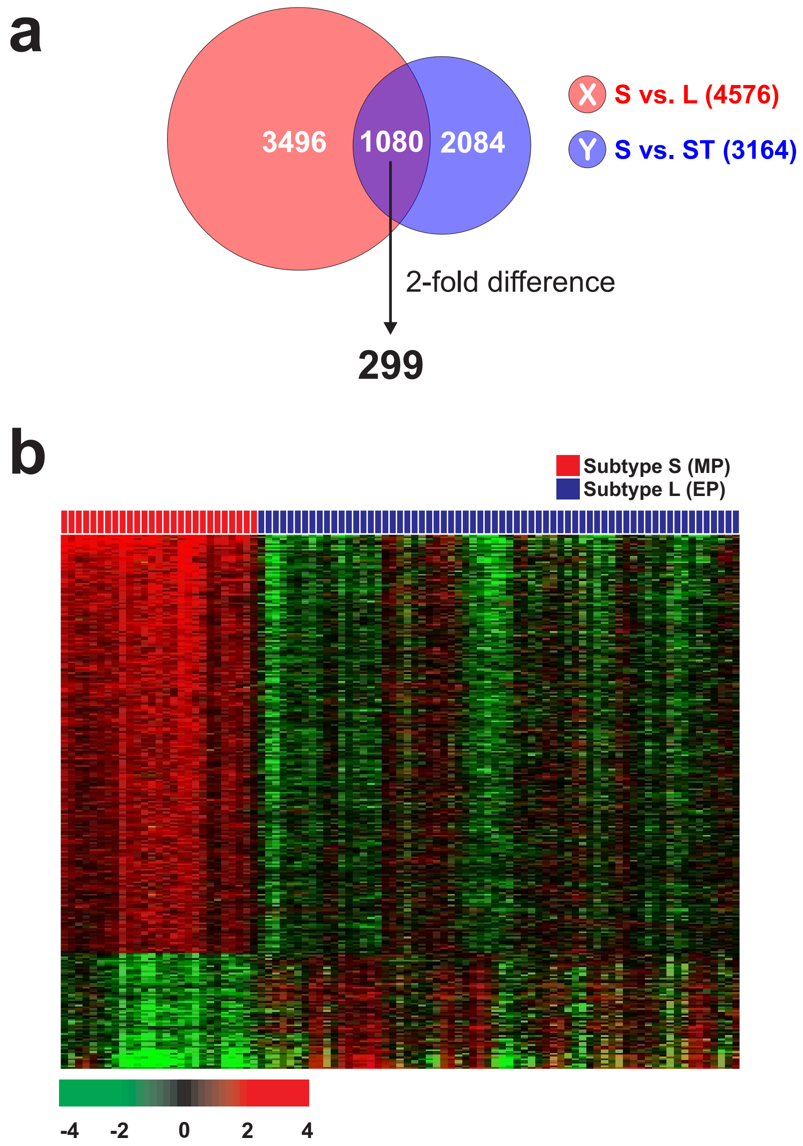

Supplementary Figure 1. Unique gene expression signature for the subtype identified by cluster S in the Korea University Guro Hospital (KUGH) cohort.

(a) Venn diagram of genes whose expression is specific to the subtype S when compared with the subtype L and with surrounding normal tissues. A Student's *t*-test was applied to gene expression data from 2 groups of samples, and differential gene expression was considered statistically significant if their *P* value was less than 0.001. This stringent significance threshold was used to limit the number of false-positive findings. S, subtype S; L, subtype L; ST, surrounding tissues in the stomach.

(b) Expression patterns of selected genes. The data are presented in a matrix format in which each row represents an individual gene and each column represents a tissue sample. Each cell in the matrix represents the expression level of a gene feature in an individual tissue sample. The red and green coloring in the cells reflects relatively high and low expression levels, respectively, as indicated in the scale bar (log2 transformed scale). MP, mesenchymal phenotype; EP, epithelial phenotype.

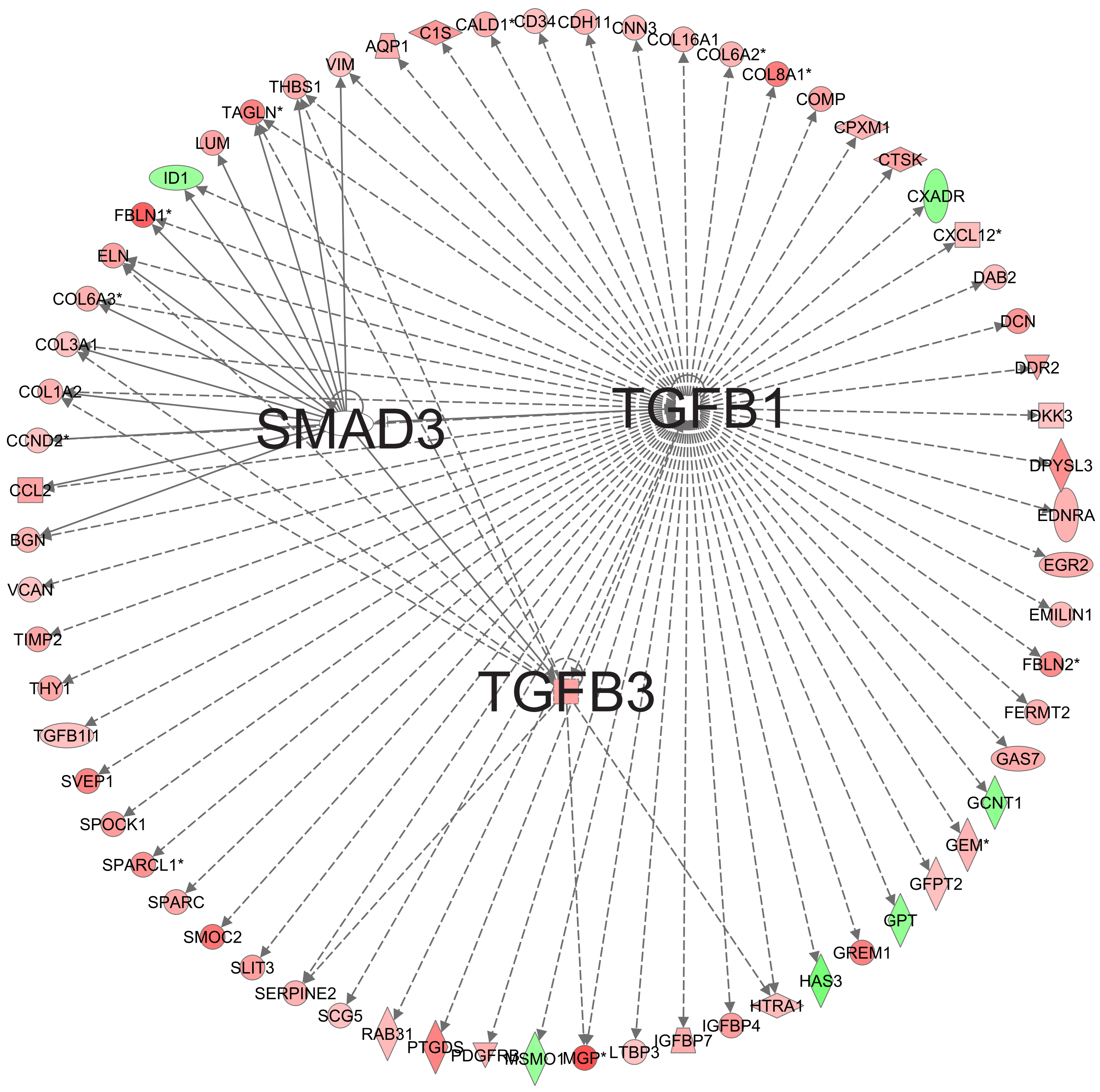

Supplementary Figure 2. Over-represented gene networks in cluster S.

Ingenuity Pathway Analysis software revealed that networks of genes considerably associated with *TGFB1, TGFB3, and SMAD3* were significantly activated in cluster S. Upregulated and downregulated genes in cluster S are indicated by red and green, respectively. The lines and arrows represent functional and physical interactions and directions of regulation from the literature

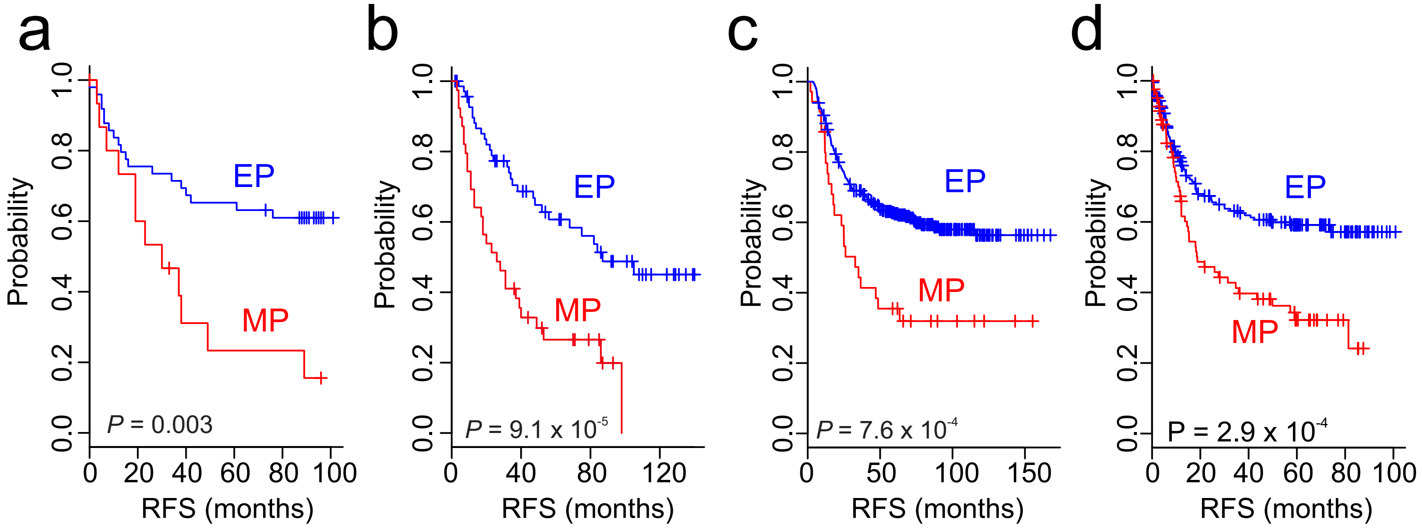

Supplementary Figure 3. Significant difference of recurrence free survival between MP and EP subtypes in validation cohorts.

Kaplan-Meier plots of recurrence-free survival (RFS) in patients with EP or MP subtype gastric cancer predicted by BCCP in the YUSH cohort **(a)**, KUCM cohort **(b)**, SMC cohort **(c)**, and ACRG **(d)**. P values were obtained using the log-rank test. The + symbols in the panels indicate censored data.

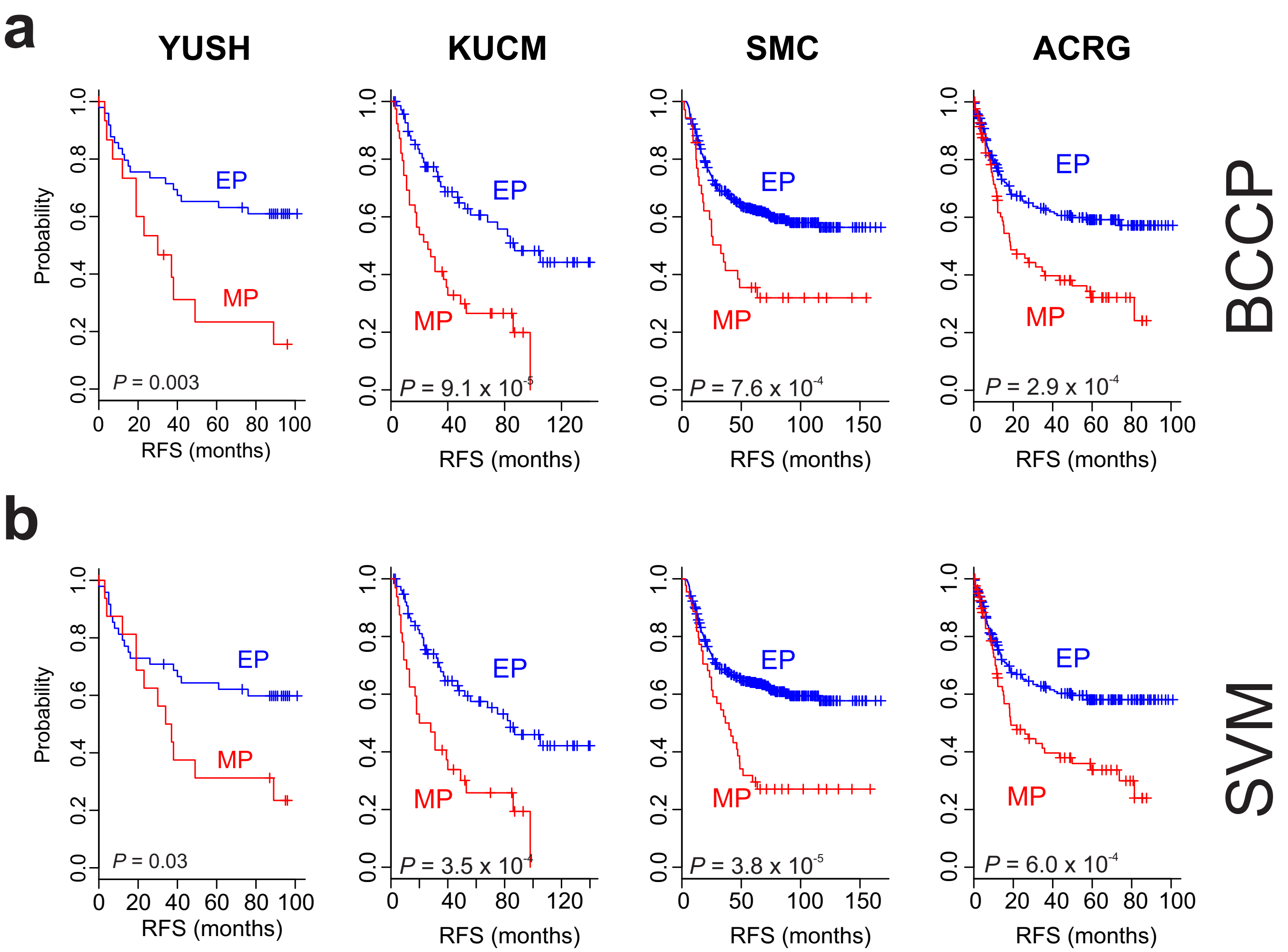

c

YUSH

BCCP \ SVM	EP	MP
EP	46	3
MP	2	14

Kappa = 0.79
P = 1.27 x 10^{-10}

KUCM

BCCP \ SVM	EP	MP
EP	70	0
MP	7	32

Kappa = 0.85
P = 2.2 x 10^{-16}

SMC

BCCP \ SVM	EP	MP
EP	379	18
MP	9	26

Kappa = 0.62
P = 2.2 x 10^{-16}

ACRG

BCCP \ SVM	EP	MP
EP	206	10
MP	12	72

Kappa = 0.82
P = 2.2 x 10^{-16}

Supplementary Figure 4. Concordant outcomes of two prediction models (BCCP and SVM).

(a, b) Two different prediction algorithms (BCCP and SVM) were applied to same gene expression data to stratify patients in three cohorts.
(c) Kappa statistics were applied to estimate concordance of two predicted outcomes.

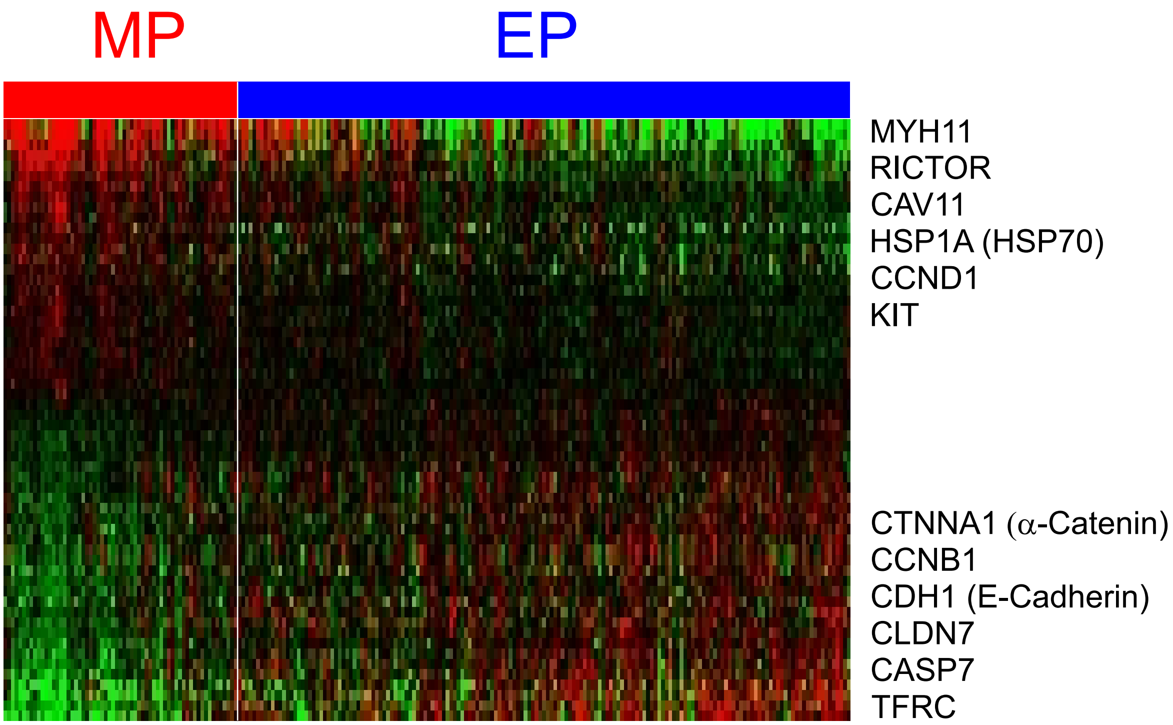

Supplementary Figure 5. Protein features significantly associated with the mesenchymal phenotype (MP) subtype of gastric cancer in TCGA cohort.

Fifty-eight protein features were significantly ($P < 0.001$) associated with genomic subtypes of gastric cancer ($P < 0.001$). The MP subtype was associated with elevated expression of MYH11, RICTOR, CAV11, HSP70, CCND1, and KIT and reduced expression of CTNNA1. The data are presented in a matrix format in which each row represents an individual protein and each column represents a tissue sample. Each cell in the matrix represents the expression level of a protein feature in an individual tissue sample. The red and green coloring in the cells reflects relatively high and low expression levels respectively.

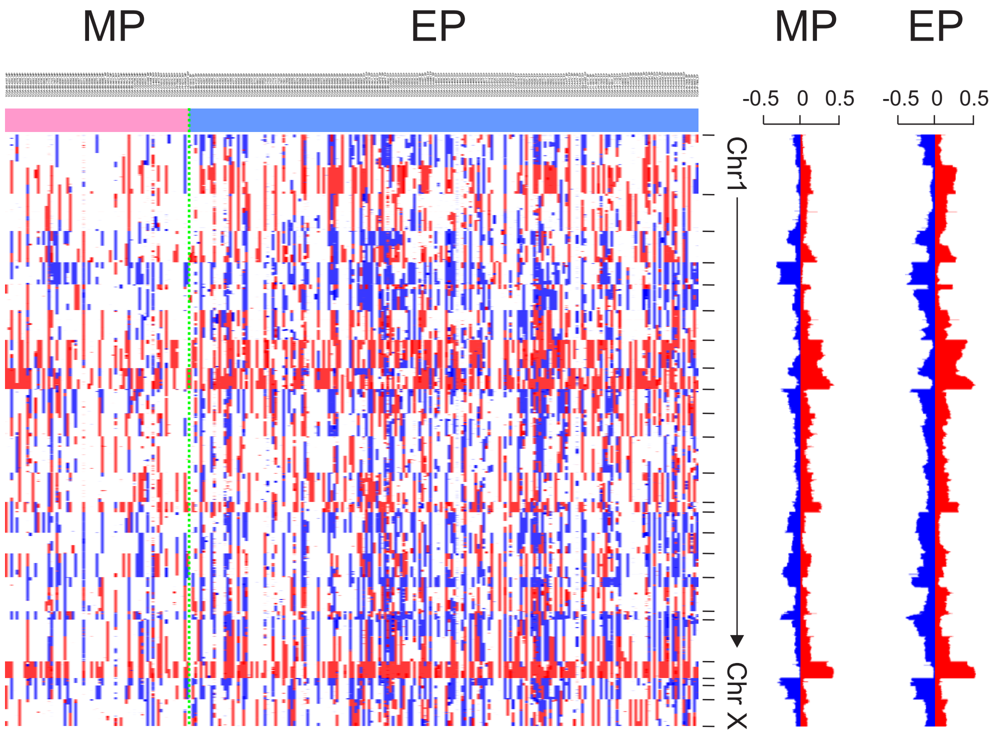

Supplementary Figure 6. Significant copy number alterations in MP and EP subtypes of gastric cancer.

The heatmap of significant focal copy number alterations that were identified from segmented data using GISTIC 2.0. is shown.

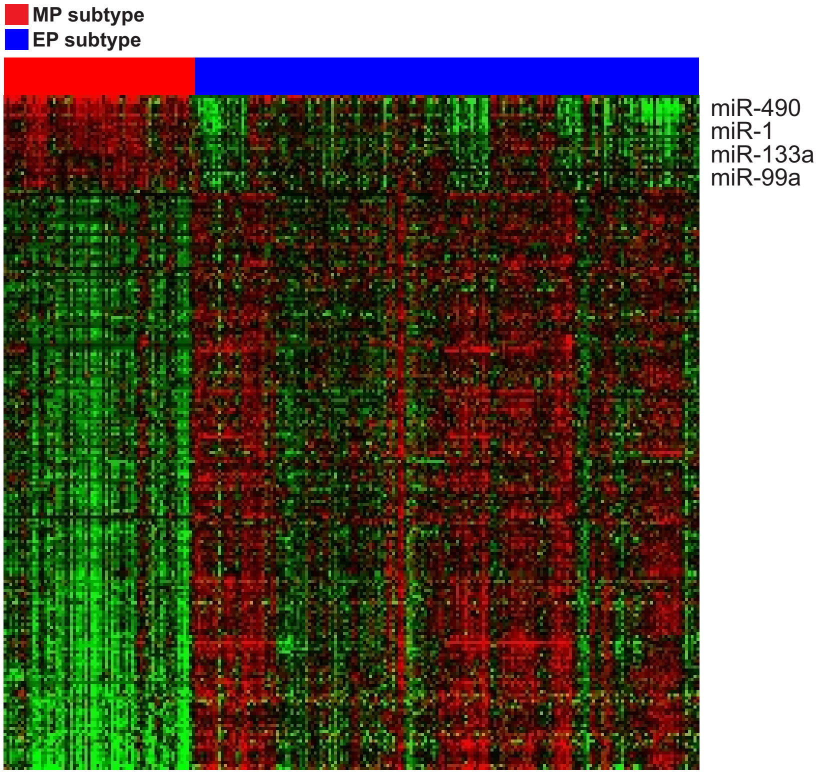

Supplementary Figure 7. Expression patterns of miRNAs significantly associated with two subtypes (MP and EP) in TCGA cohort.

Expression of 220 miRNAs was significantly (*P* < 0.001; > 1.5-fold difference in expression level) associated with MP and EP subtypes of gastric cancer. miRNAs were ranked according to average expression ratios between MP and EP subtype tumors.

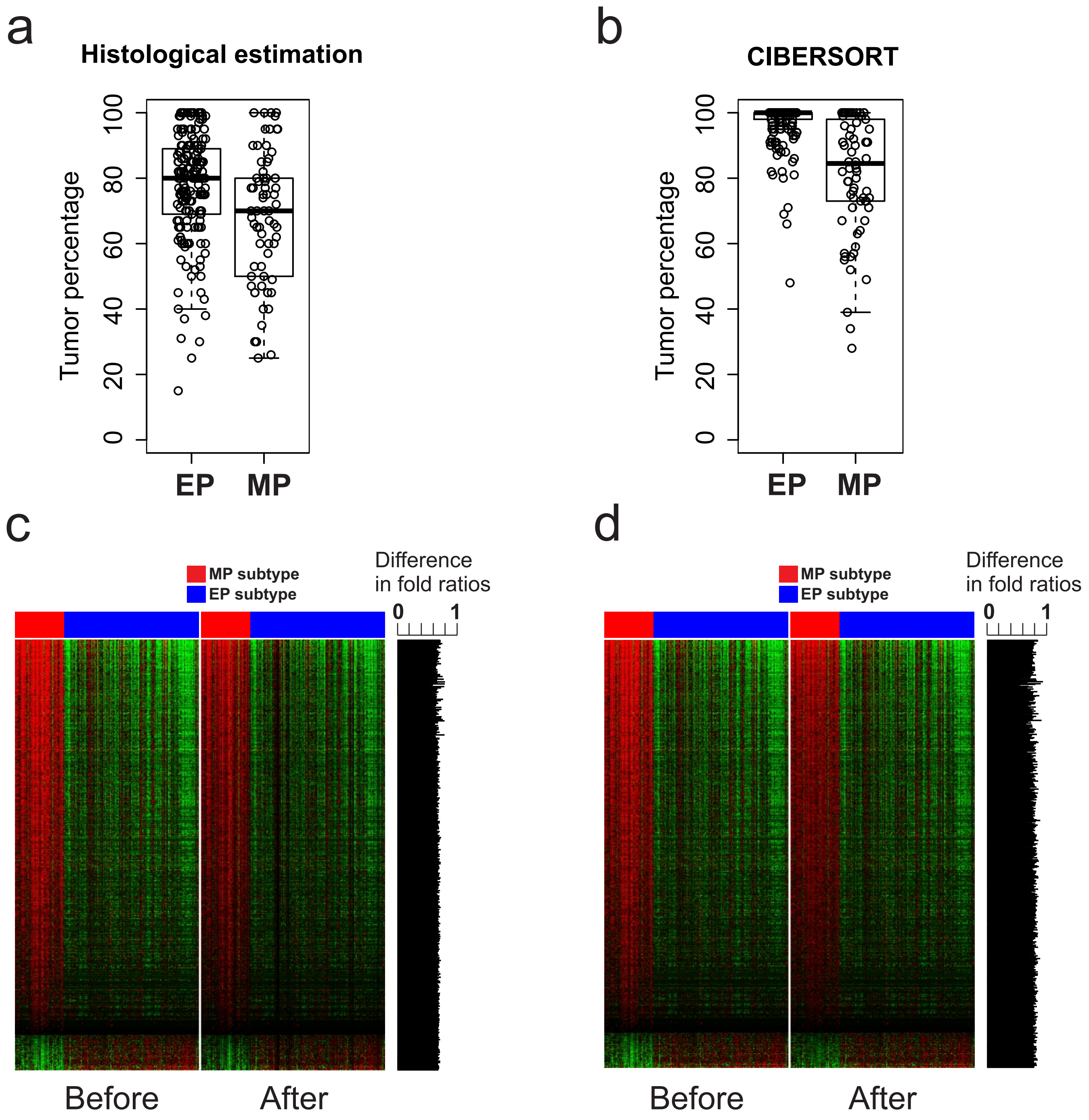

Supplementary Figure 8. Effect of non-tumor cells in gene expression data from gastric tumors in TCGA cohort.

(a) Estimated percentage of tumor cells by CIBERSORT that quantifies the relative levels of distinct cell types within a complex gene expression admixture like tumor mass.
(b) Histologically measured percentage of tumor cells.
(c,d) Heatmaps of before and after re-normalization of gene expression data: To remove contribution of non-tumor cells to gene expression data from tumor mass, gene expression data were re-normalized by multiplying tumor purity scores (percentage of tumor cells) that were estimated by CIBERSORT (**c**) or histologically measured (**d**). Barplots next to heatmap present difference of gene expression ratios between MP and EP subtypes before and after re-normalization.

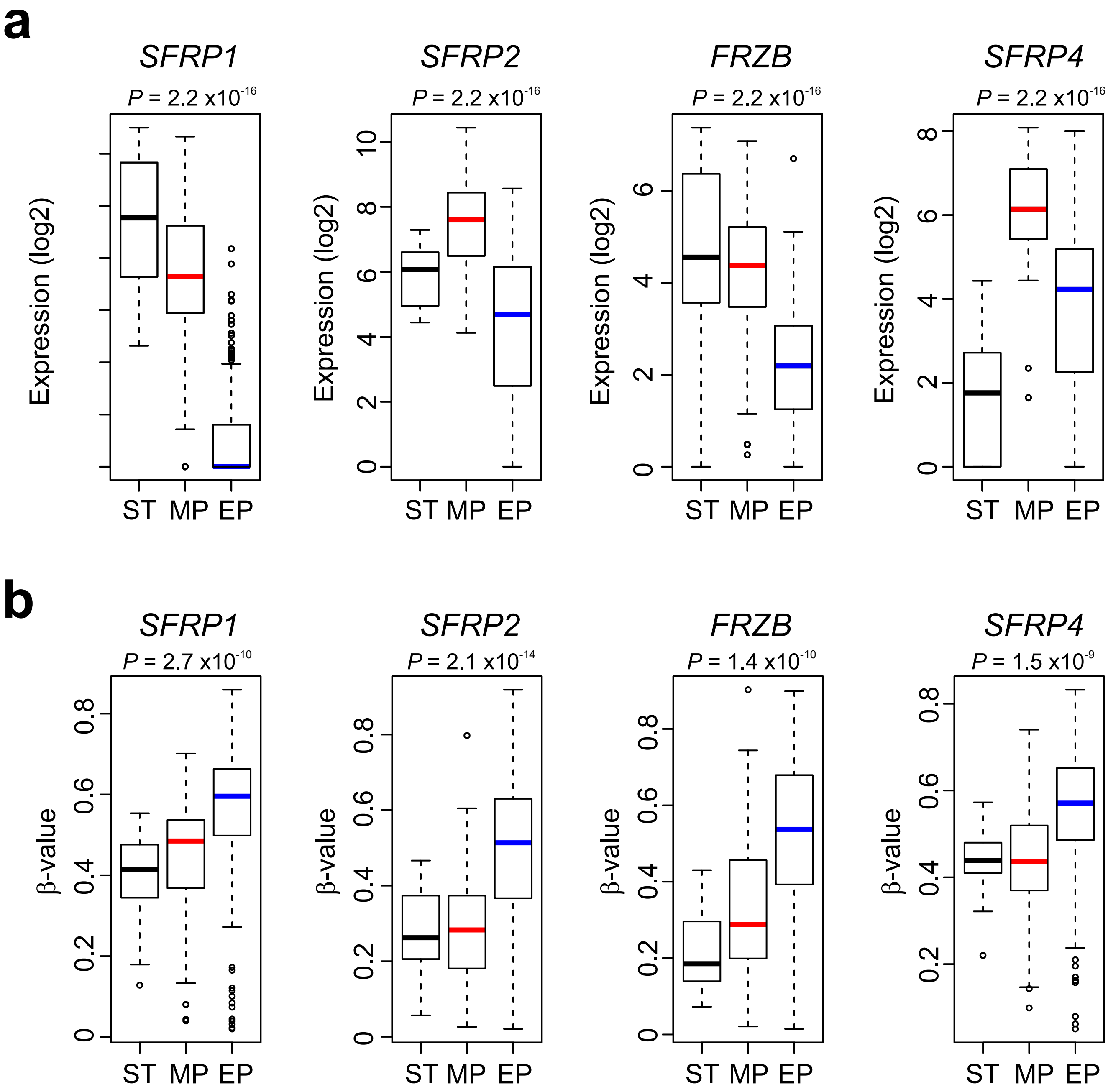

Supplementary Figure 9. Down-regulation of SFRPs is mediated by promoter methylation.

(a) Differential expression of SFRPs in 2 subtypes of gastric cancer in TCGA cohort.
(b) Differential promoter methylation of SFRPs in surrounding tissues (ST), MP, and EP subtypes of gastric cancer in TCGA cohort. P values represent the difference in expression between MP and EP subtype tumors and were determined by a Student's t-test. Colored lines indicate the median, boxes extend from the 25th to the 75th percentile and dashed error bars extend to the 10th and 90th percentiles.

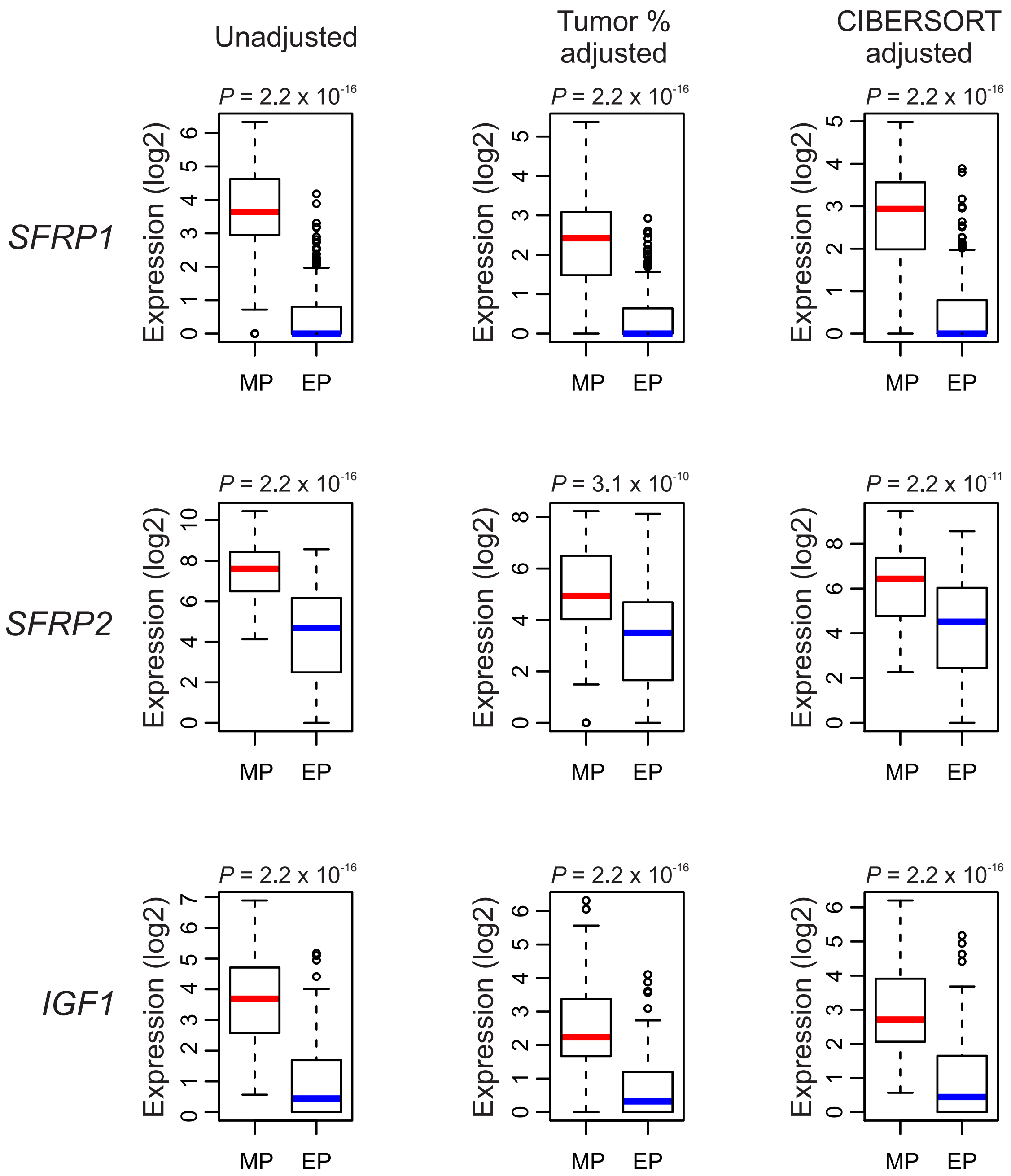

Supplementary Figure 10. Expression level of genes before and after adjustment of non-tumor cell effects.

Contribution of expression from non-tumor cells were adjusted by multiplying predicted tumor cell percentage from CIBERSORT or histologically defined tumor percentage.

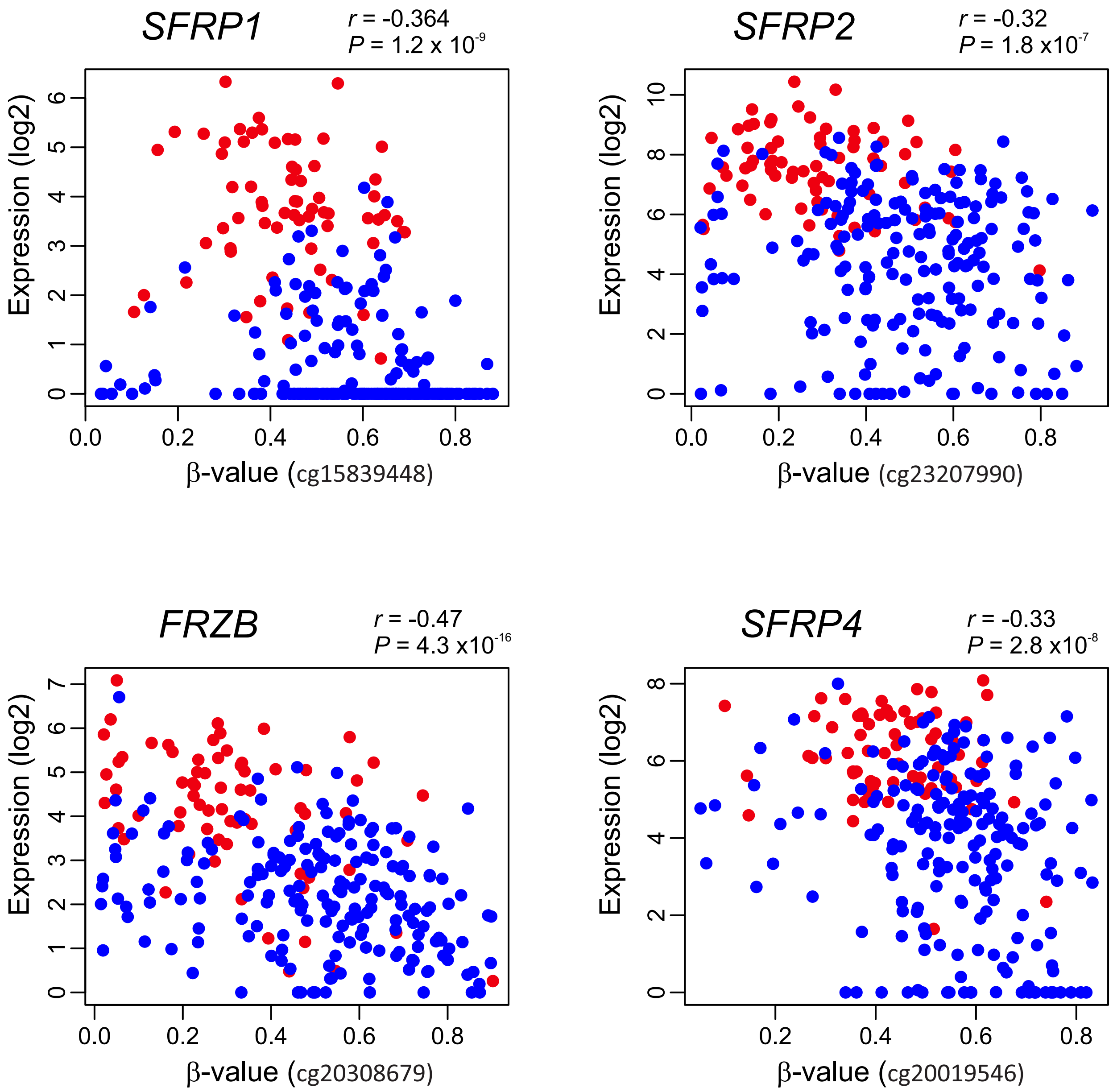

Supplementary Figure 11. Expression of SFRPs is inversely correlated with promoter methylation.

Correlation between mRNA expression and promoter methylation (β-value) was estimated by Pearson correlation method. Each tissue was colored by subtypes (MP: red, EP:blue). Probe ID were indicated in parenthesis.

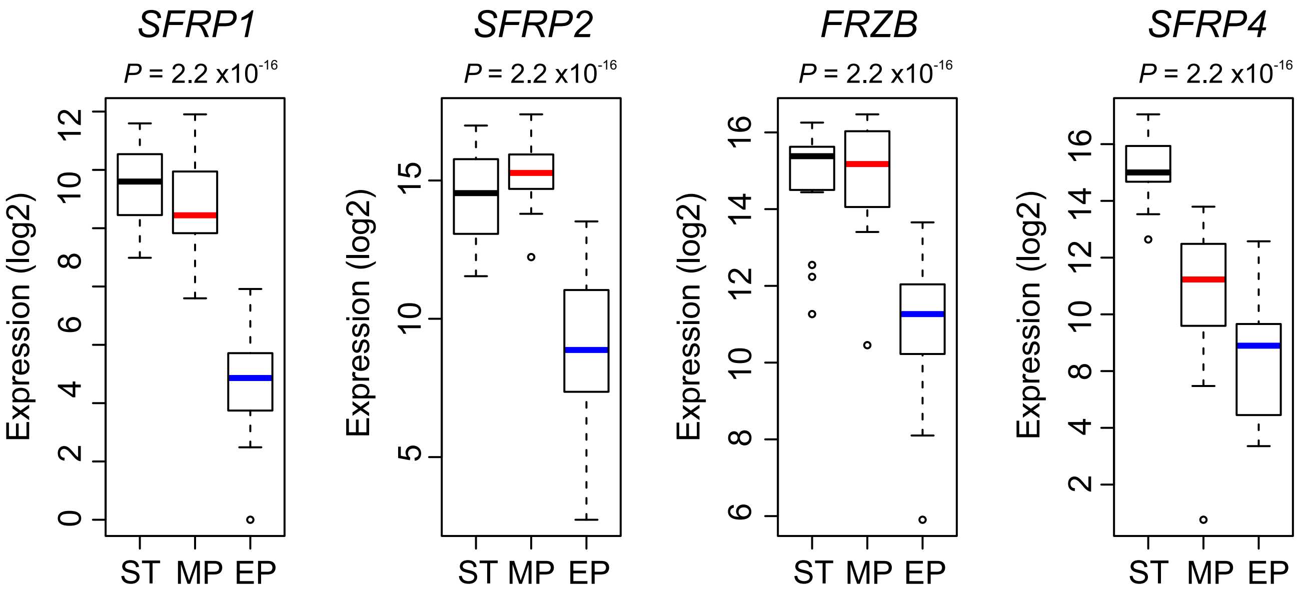

Supplementary Figure 12. Validation of down-regulation of SFRPs in YUSH cohort.

Expression of SFRPs in 2 subtypes of gastric cancer and surrounding normal tissues were measured by qRT-PCR. in YUSH cohort. P values represent the difference in expression between MP and EP subtype tumors and were determined by a Student's t-test. Colored lines indicate the median, boxes extend from the 25th to the 75th percentile and dashed error bars extend to the 10th and 90th percentiles.

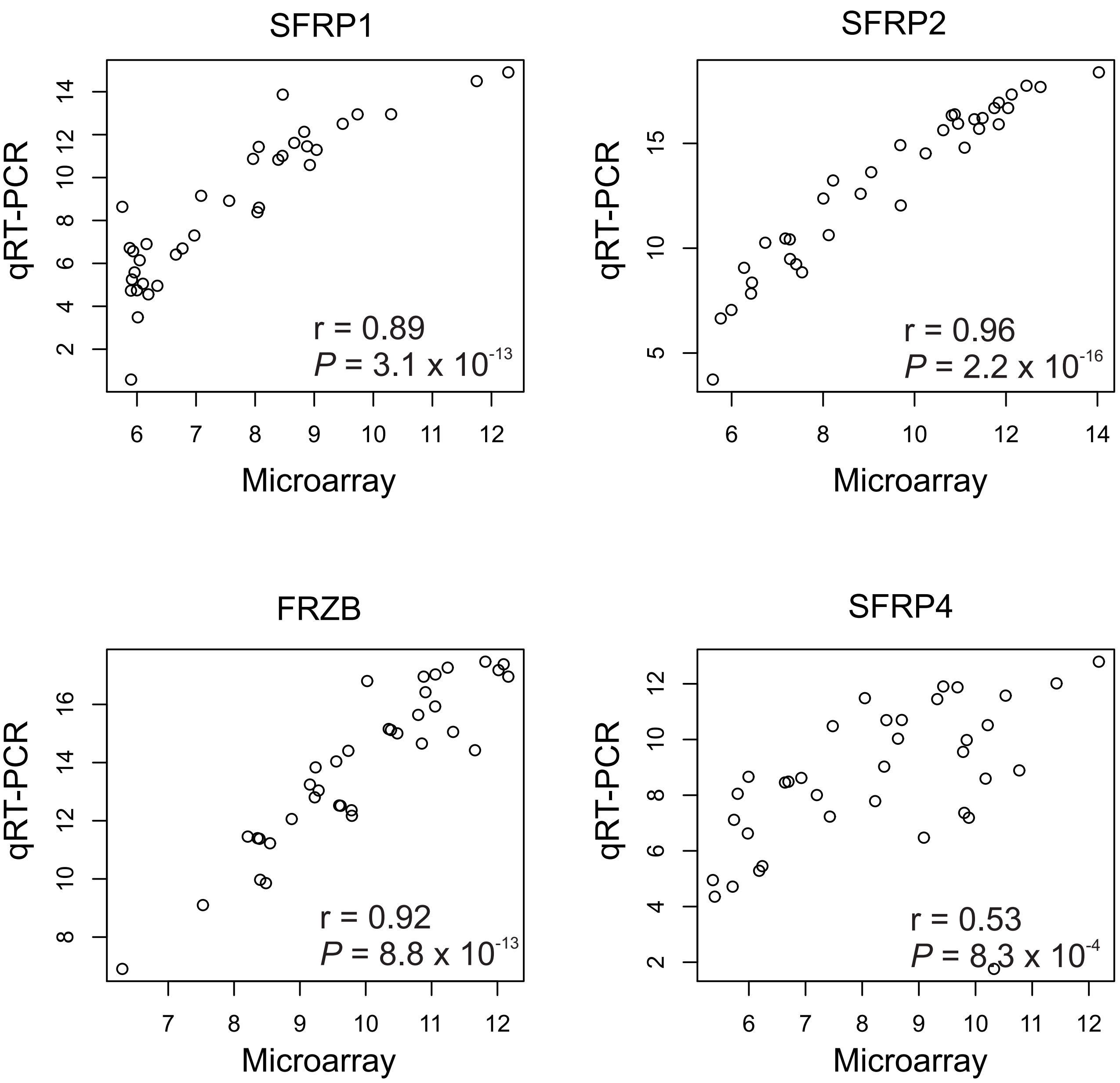

Supplementary Figure 13. Correlation between mRNA expression measurements by microarray and qRT-PCR experiments in YUSH cohort.

Expression of 4 SFRP family members were measured by two experimental methods (microarray and qRT-PCR)

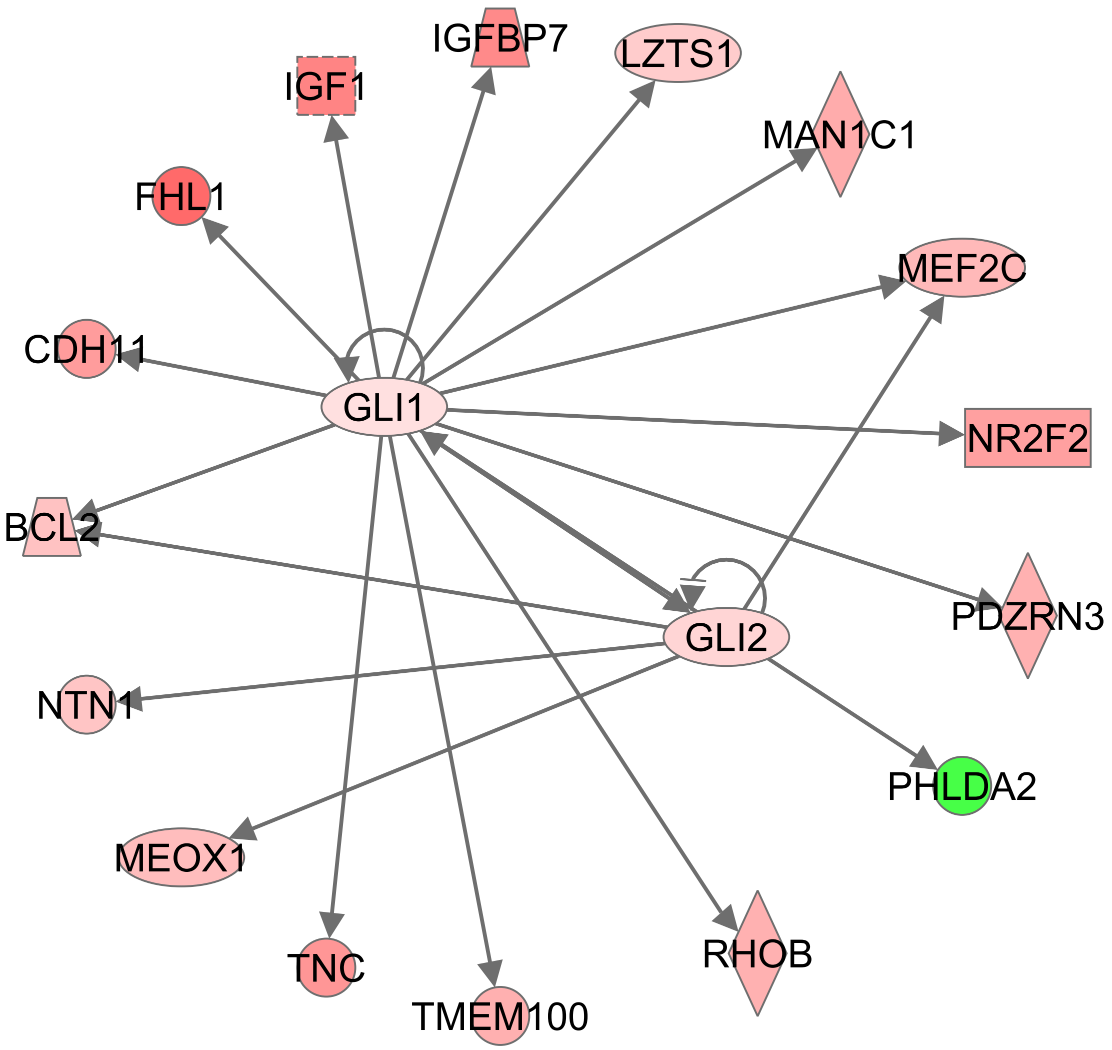

Supplementary Figure 14. Activation of hedgehog pathway in MP subtype.

Analysis using Ingenuity Pathway Analysis software revealed networks of genes considerably associated with *GLI1* and *GLI2* in conserved gene expression data from 4 cohorts. Upregulated and downregulated genes in the MP subtype are indicated by red and green, respectively. The lines and arrows represent functional and physical interactions and directions of regulation from the literature.

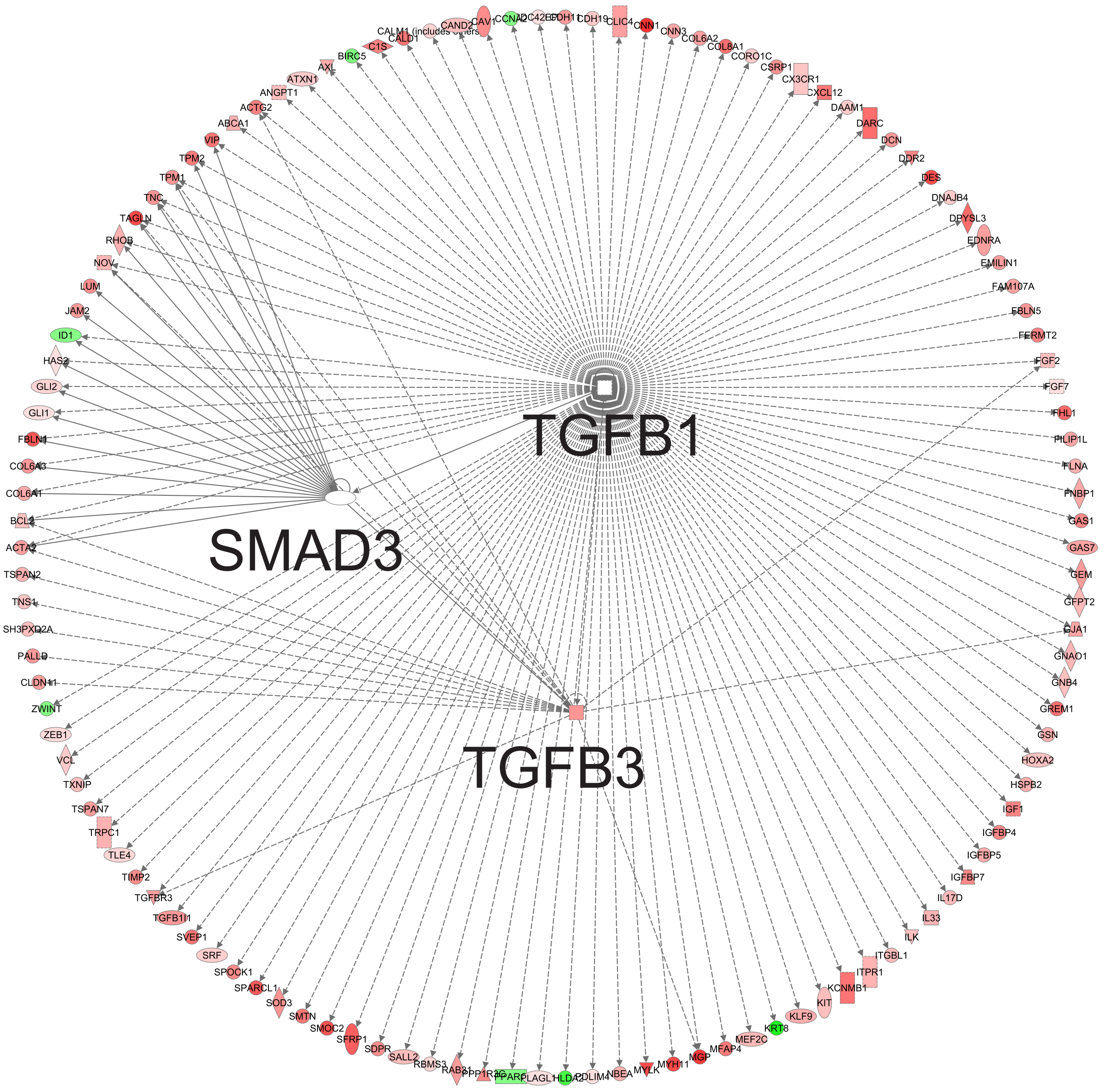

Supplementary Figure 15. **Activated TGF-β pathway in MP subtype.**

Analysis using Ingenuity Pathway Analysis software revealed that networks of genes considerably associated with *TGFB1, TGFB3, and SMAD3* were significantly activated in MP subtype tumors. Upregulated and downregulated genes in MP subtype are indicated by red and green, respectively. The lines and arrows represent functional and physical interactions and directions of regulation from the literature.

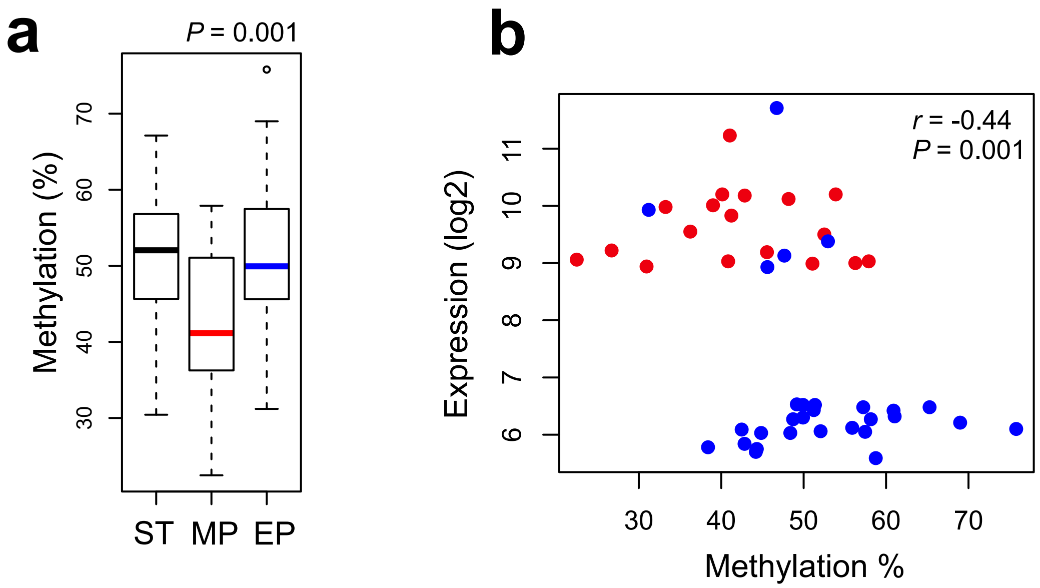

Supplementary figure 16. Validation of association between promoter methylation and expression of IGF1 in YUSH and KUCM cohorts.

(**a**) Promoter methylation of the IGF1 gene in each subtype of gastric cancer and ST in the selected samples from YUSH and KUCM cohorts. Colored lines indicate the median, boxes extend from the 25th to the 75th percentile, and dashed error bars extend to the 10th and 90th percentiles. Differences between MP and EP groups were significant (*P* = 0.001 by Student's t-test). (**b**) Correlation between mRNA expression and promoter methylation, estimated using Pearson correlation.

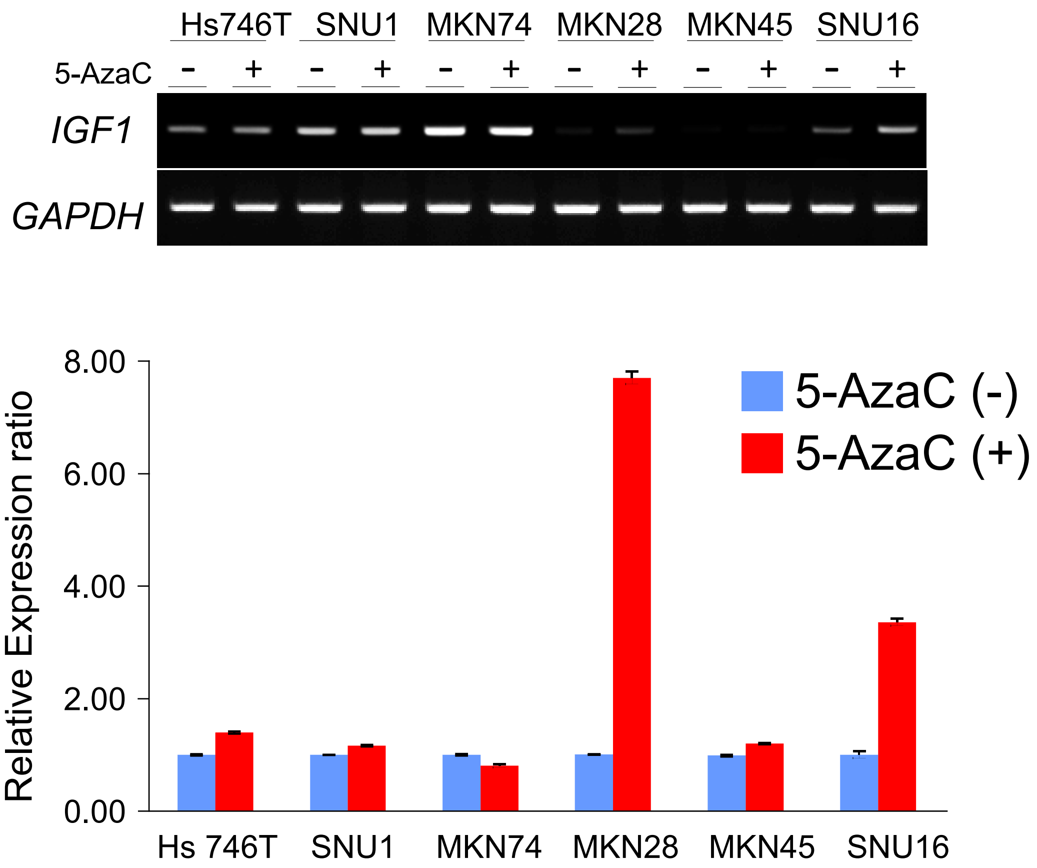

Supplementary Figure 17. **Regulation of IGF1 expression by promoter methylation in GC cell lines.**

Expression of IGF1 was restored in EP-like MKN28 and SNU16 cells by 5-AzaC treatment, as measured by aRT-PCR, GAPDH serves as the control. Error bars indicate s.e.m.

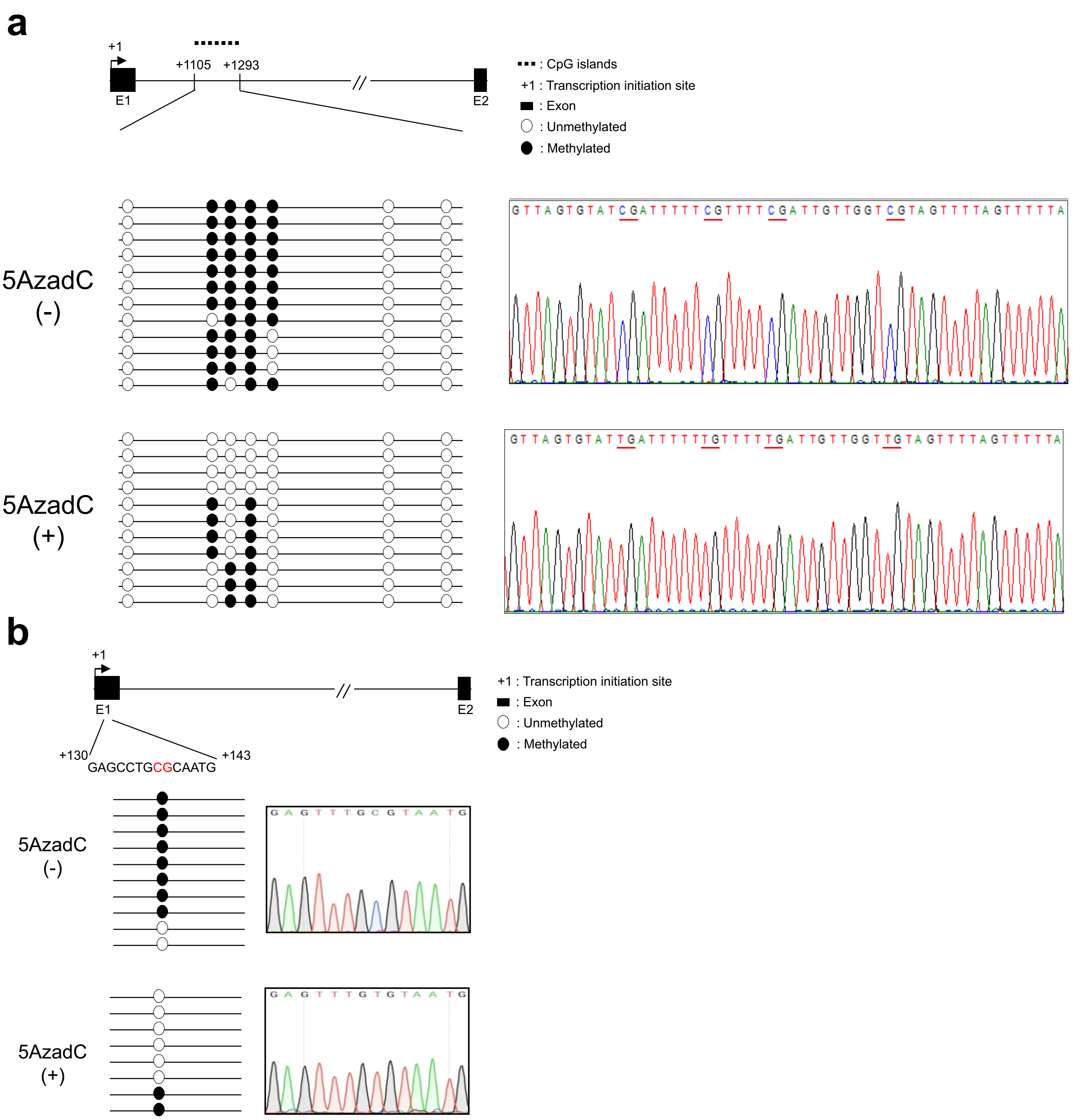

Supplementary Figure 18. DNA methylation of IGF1 in MKN28 gastric cancer cell line.

(a) TOP: Schematic view of the IGF1 CpG sites between +1105 to +1293 nucleotides from the transcription initiation site investigated. BOTTOM: Bisulfite sequencing analysis of the IGF1 CpG sites in EP subtype MKN28 cell. For DNA demethylation, 5-AzadC was treated at concentrations of 2 µM for 72 hrs. A representative sequencing data were shown.
(b) TOP: Schematic view of the IGF1 CpG sites between +130 to +143 nucleotides from the transcription initiation site investigated. BOTTOM: Bisulfite sequencing analysis of the IGF1 CpG sites in EP subtype MKN28 cell. For DNA demethylation, 5-AzadC was treated at concentrations of 2 µM for 72 hrs. A representative sequencing data were shown.

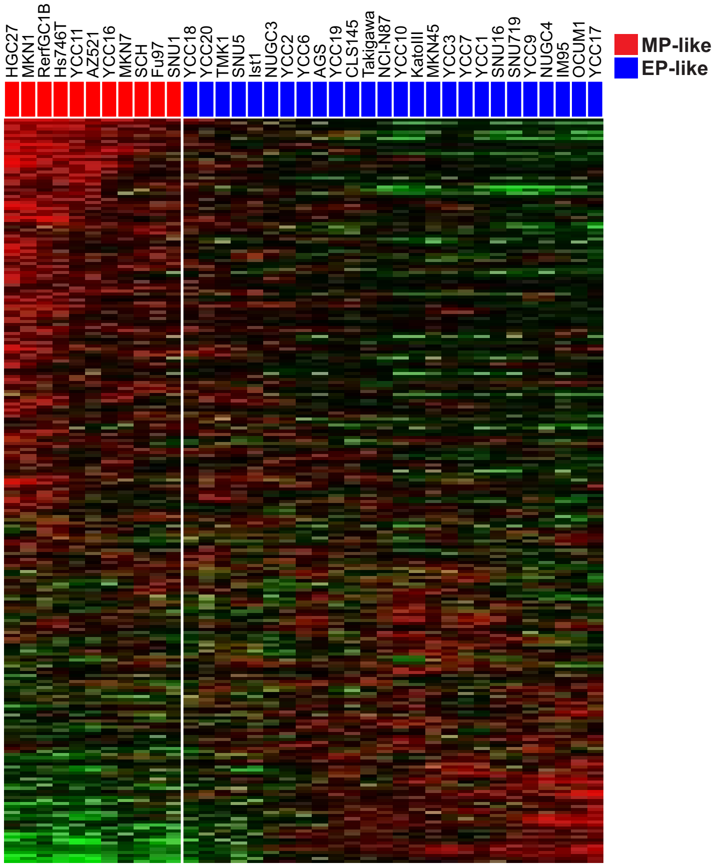

Supplementary Figure 19. **Expression patterns of MP signature in 37 gastric cancer cell lines.**

Expression patterns of MP signature in gastric cancer cell lines. Bayesian compound covariate predictor was applied to gene expression data from 37 gastric cancer cell lines (GSE22183) and probability of mesenchymal phenotype was generated as described in Fig. 2. MKN74 and MKN28 were not included from analysis because their expression data were not available from GSE22183 data set. Of 299 genes in MP signature, 244 genes were presented in gene expression data from cell lines. The data are presented in a matrix format in which each row represents an individual gene and each column represents a tissue sample. Each cell in the matrix represents the expression level of a gene feature in an individual tissue sample. The red and green coloring in the cells reflects relatively high and low expression levels, respectively, as indicated in the scale bar (log2 transformed scale). MP, mesenchymal phenotype; EP, epithelial phenotype.

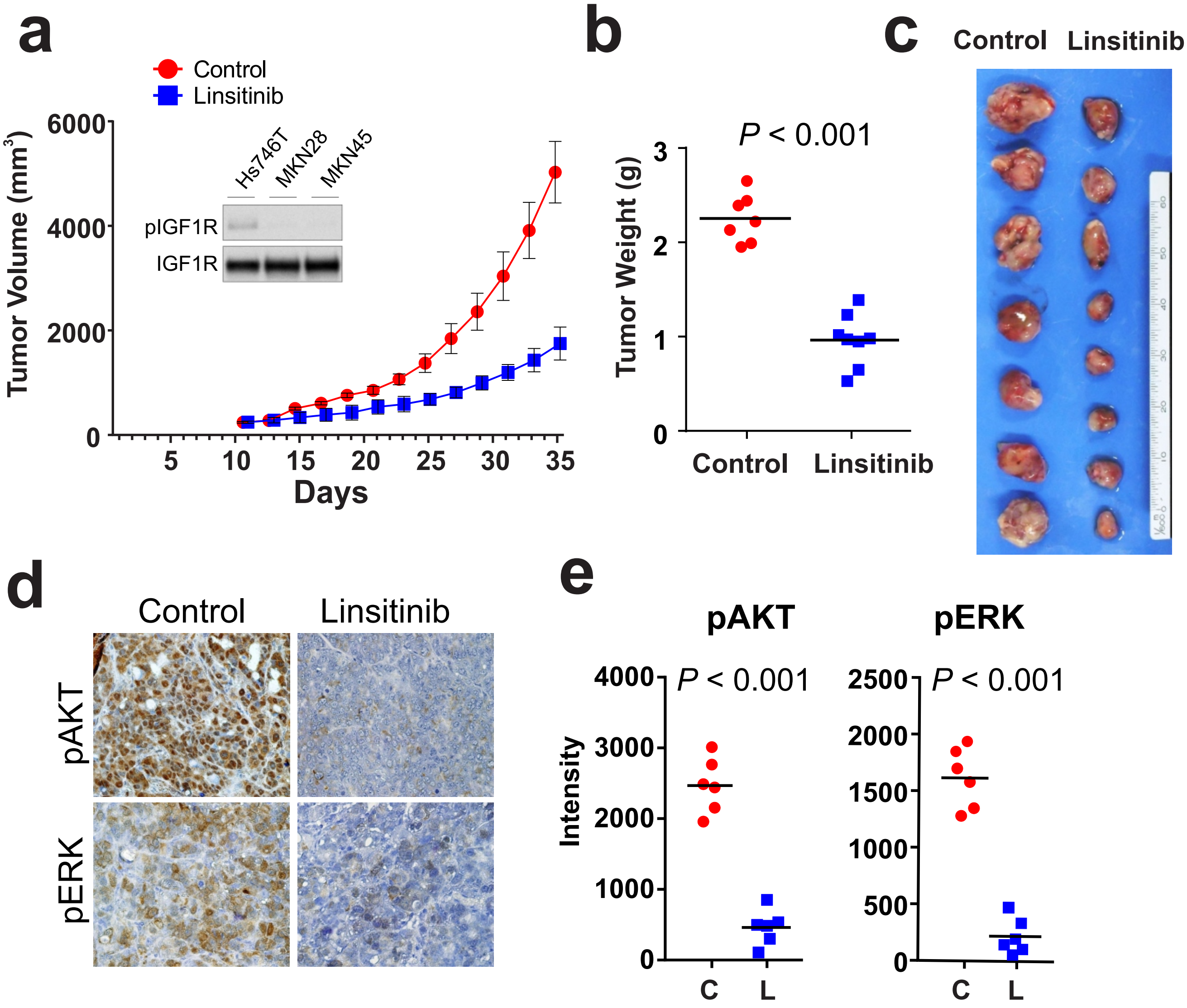

Supplementary Figure 20. Therapeutic effect of IGF1R inhibition in Hs746T cell.
(a) Growth of Hs746T-derived xenograft tumors in mice treated with linsitinib or vehicle control. Hs746T cells were xenografted subcutaneously into the flanks of mice. At 10 days after xenografting, linsitinib or control tartaric acid was orally administrated to mice. Tumor volume was measured on the indicated days.
(b) Tumor weight after treatment of linsitinib. Twenty-five days after linsitinib treatment, mice were killed and tumor weights were measured (n = 7 or 8 per treatment). Data are presented with means. P values were obtained by Student's t-test.
(c) Tumors harvested after treatment of linsitinib or control vehicle.
(d) Representative immunohistochemical staining of pAKT1 and pERK1/2 in Hs746T xenograft tumors treated with linsitinib and control vehicle.
(e). Phosphorylation levels of AKT1 and ERK1/2 were compared among tumors treated with linsitinib or control vehicle. P values were obtained by Student's t-test.

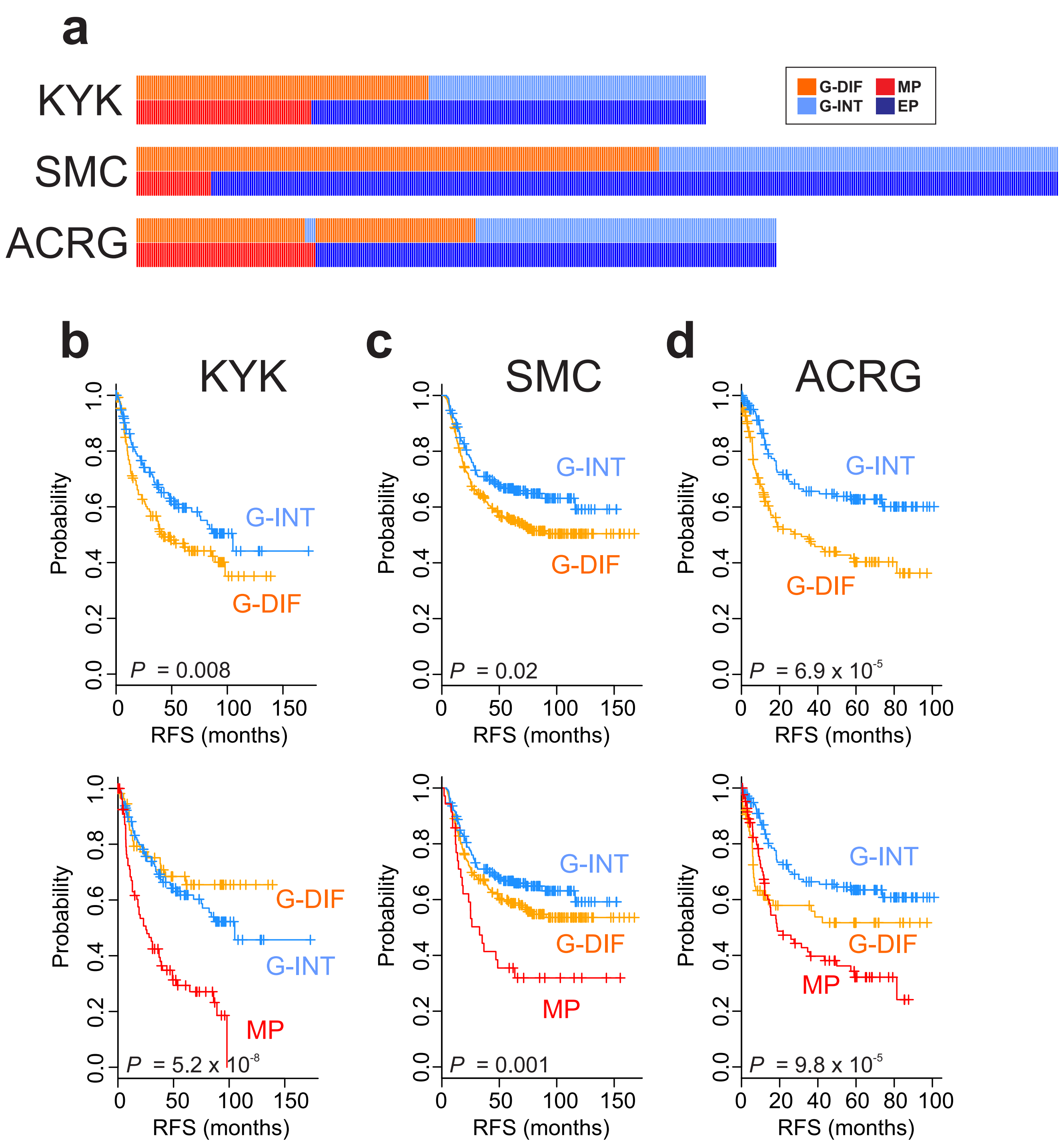

Supplementary Figure 21. MP subtype is related to G-DIF subtype but clinically distinct from G-DIF tumors.

(**a**) Membership of GC tumors in two different subtype categories. Patients were stratified according to two different prognostic gene expression signatures (G-DIF/G-INT signature and GIST signature). Patients in Korea University, Yonsei University, and Kosin University were pooled together and renamed as KYK cohort for analysis.
(**b, c, d**) Kaplan-Meier plots of recurrence free survival (RFS) of patients stratified by G-DIF/G-INT signature alone (top) and combination of G-DIF/G-INT signature and GIST signature (bottom) in KYK (**b**), SMC (**c**), and ACRG (**d**) cohorts.

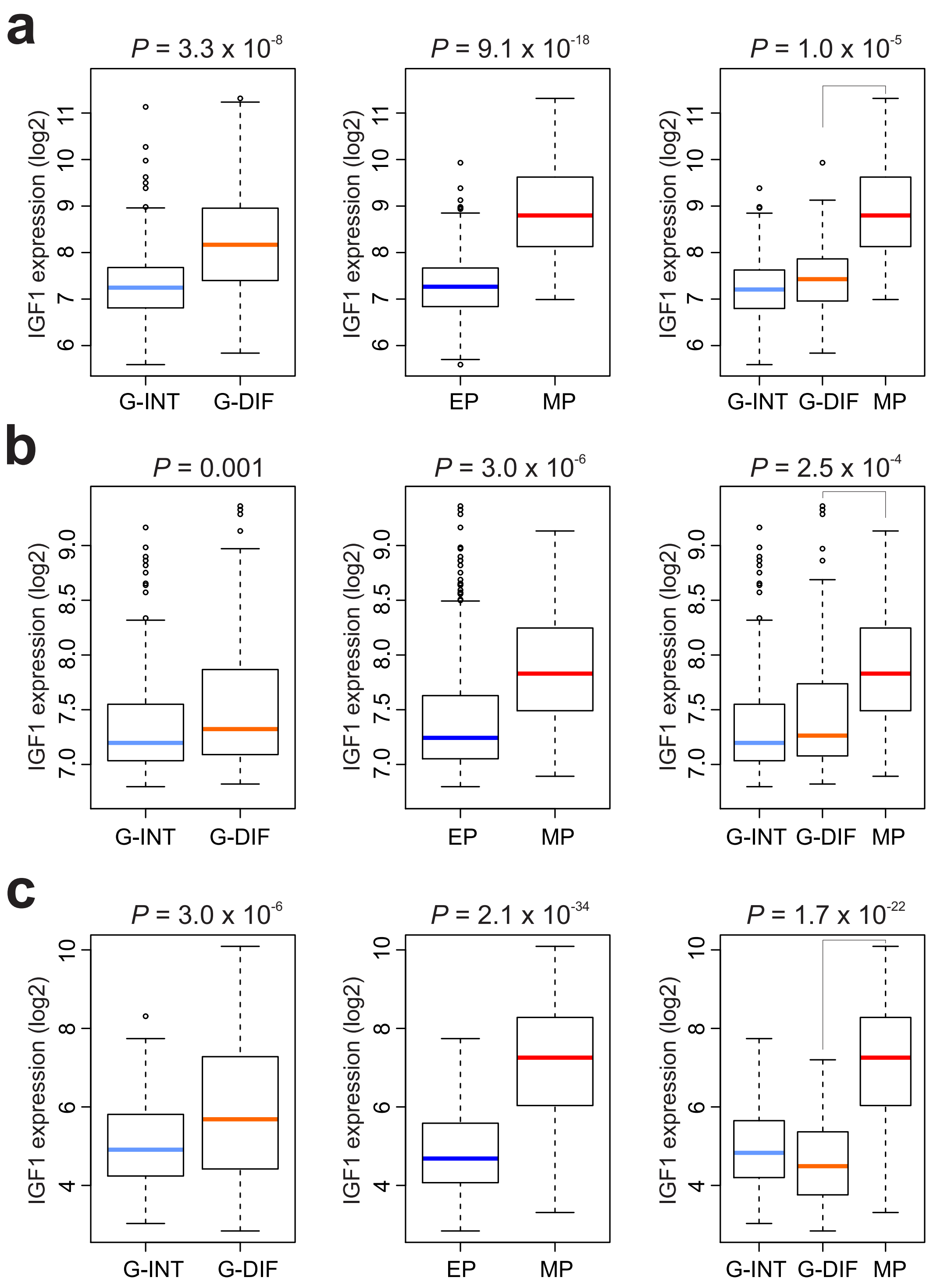

Supplementary Figure 22. Expression of IGF1 in subtypes stratified by two different prognostic gene expression signatures (G-DIF/G-INT and GIST signatures for first and second columns) or in combination of two signatures (third column). **(a)** KYK cohort, **(b)** SMC cohort, and **(c)** ACRG cohort.

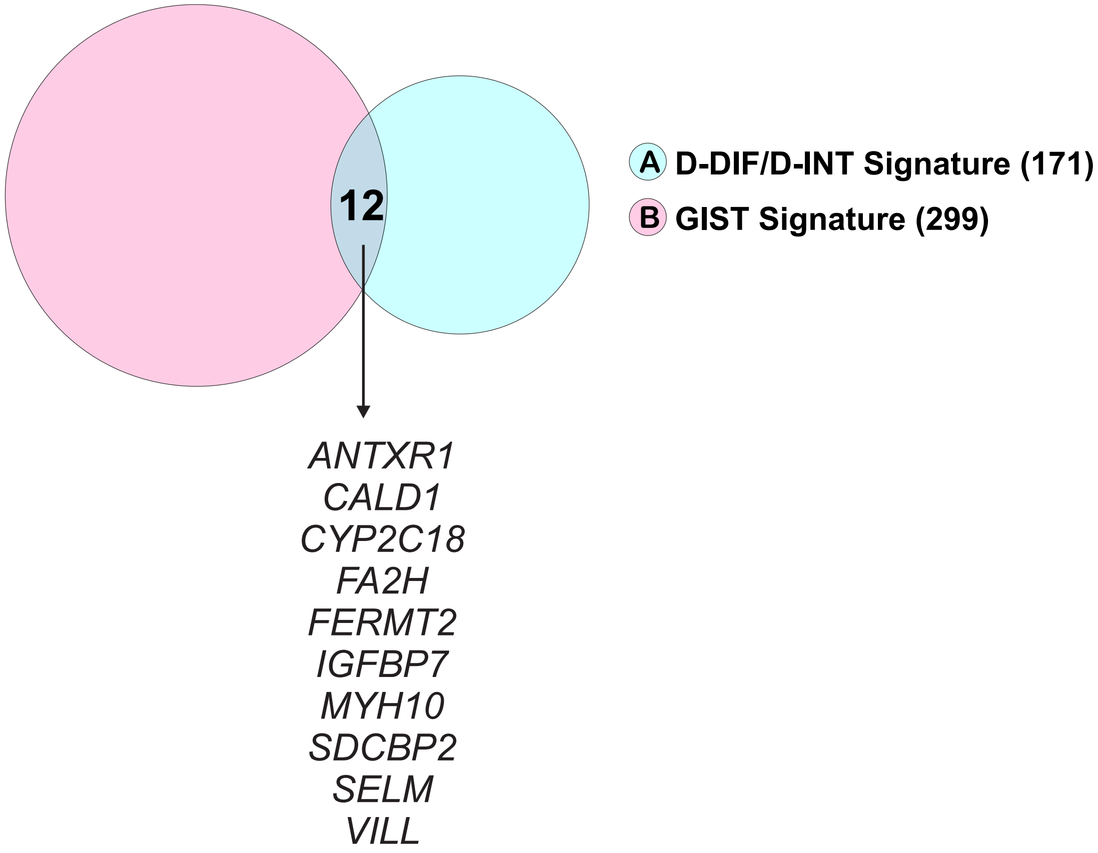

Supplementary Figure 23. Shared genes between two different prognostic signatures.

Only 12 genes were shared in G-DIF/G-INT and GIST signature.

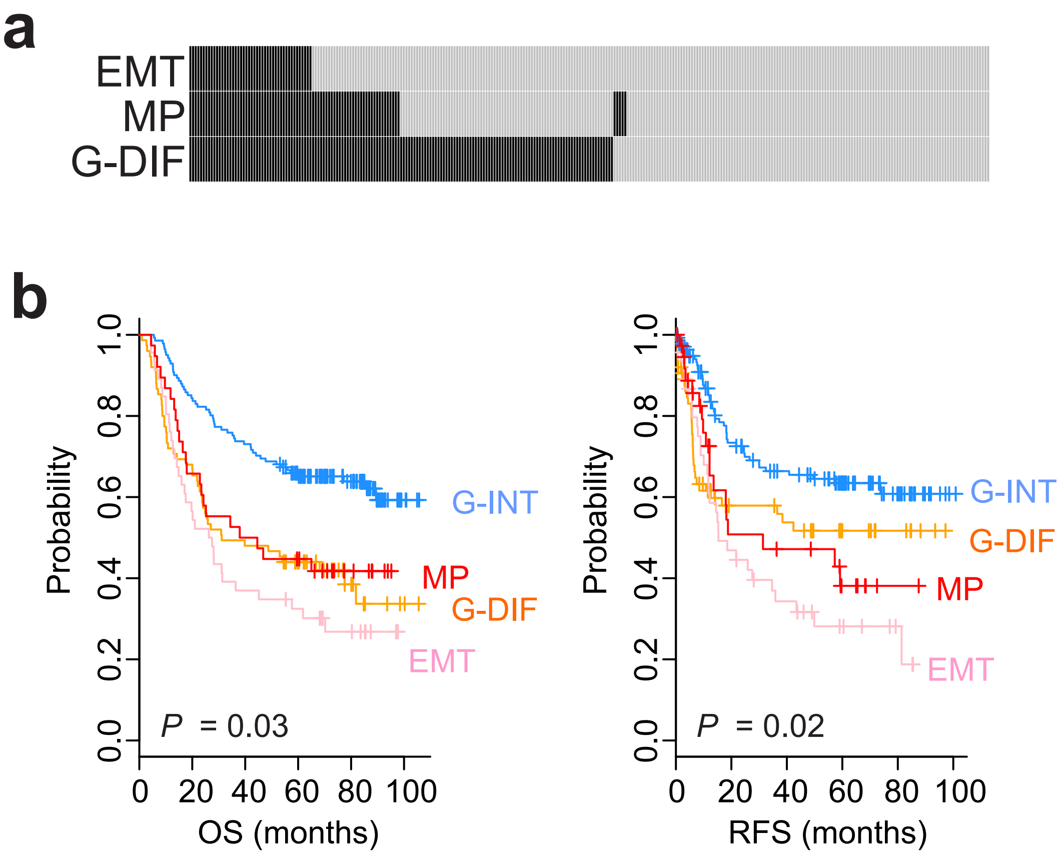

Supplementary Figure 24. Inter-relation of MP subtype with EMT and G-DIF subtype

(**a**) Membership of GC tumors in three different subtype categories. Patients in ACRG cohort were stratified according to three different prognostic gene expression signatures (G-DIF/G-INT signature, MP, and ACRG signature).
(**b**) Kaplan-Meier plots of overall survival (OS) and recurrence free survival (RFS) of patients stratified by three signature. MP subtype is subset of G-DIF while EMT is subset of MP subtype.

Supplementary Figure 25. The original full scan of immunoblot utilized in Figure 7e

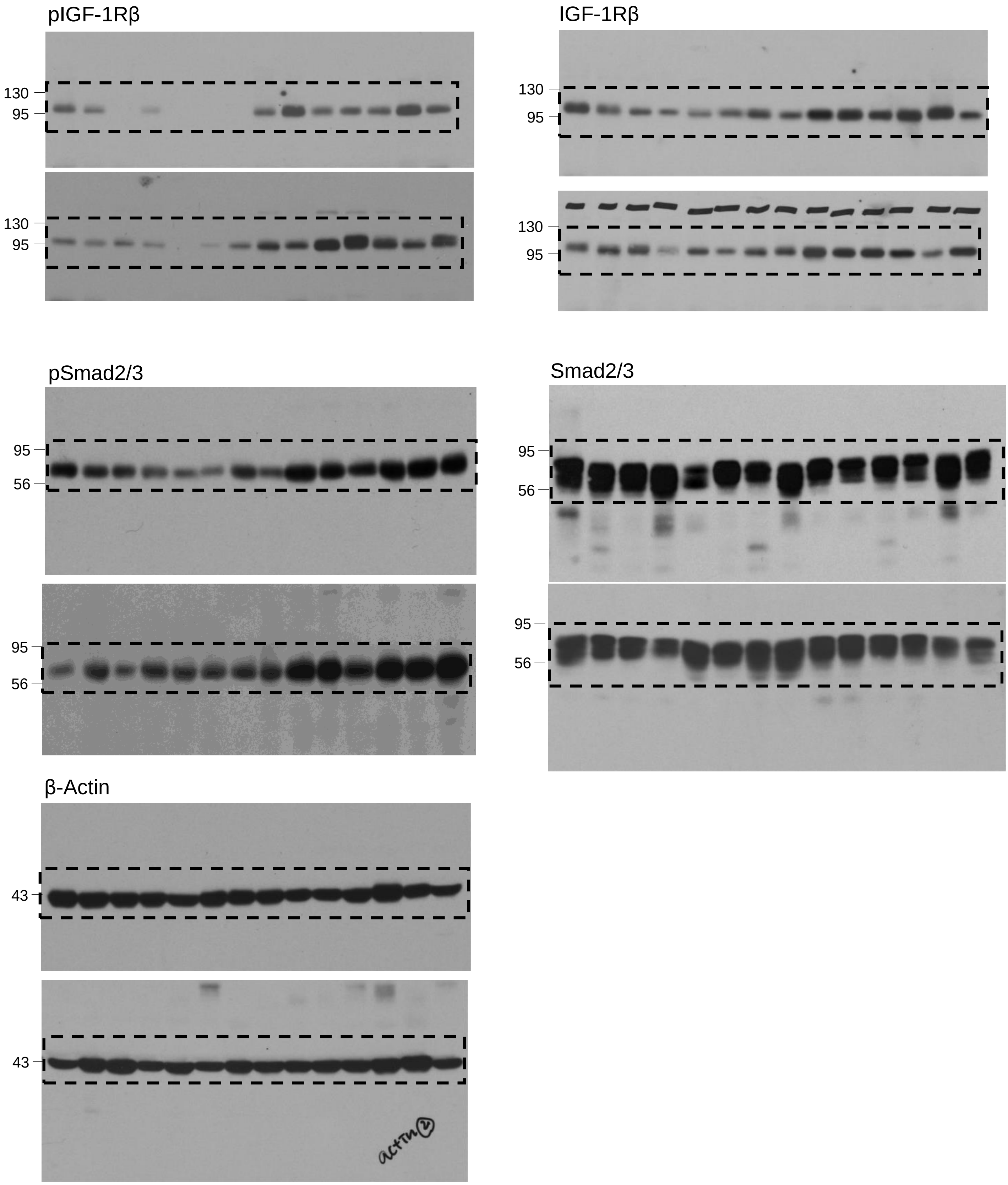

Supplementary Table 1. Clinical and pathological features of patients with gastric adenocarcinoma

Variable		KUGH cohort[a]	YUSH cohort[a]	KUCM cohort[a]	MDACC cohort[a]	SMC cohort[a]	ACRG cohort[a]
Cohort types		Exploration	Validation	Validation	Validation	Validation	Validation
No. of patients		93	65	109	40	432	300
Sex	Male	73 (78.5)	46 (70.8)	69 (63.3)	27 (67.5)	280 (64.8)	101 (33.7)
	Female	20 (21.5)	19 (29.2)	40 (36.7)	13 (32.5)	152 (35.2)	199 (66.3)
Age (years)	Median	60	63	58	58	53	64
	range	36-81	32-83	28-75	33-78	23-74	24-86
Location	Cadia	7 (7.5)	5 (7.7)	0	11 (27.5)	53 (12.3)	32 (10.6)
	Fundus	2 (2.2)	1 (1.5)	13 (11.9)	0 (0)	0 (0)	0 (0)
	Body	29 (31.2)	31 (47.7)	36 (33.0)	23 (57.5)	130 (30.1)	107 (35.6)
	Antrum	55 (59.1)	25 (38.5)	56 (51.4)	6 (15)	231 (53.5)	155 (51.5)
	Entire	0	1 (1.5)	4 (3.7)	0	17 (3.9)	6 (2)
	NA[b]	0	2 (3.1)	0	0	1 (0.2)	1 (0.3)
AJCC stage[c]	I	11 (11.8)	12 (18.5)	40 (36.7)	1 (2.5)	68 (15.7)	30 (10)
	II	18 (19.4)	12 (18.5)	18 (16.5)	6 (15)	167 (38.7)	97 (32.3)
	III	27 (29.0)	25 (38.5)	36 (33.0)	12 (30)	130 (30.1)	96 (32)
	IV	36 (38.7)	16 (24.5)	15 (13.8)	21 (52.5)	67 (15.5)	77 (25.7)
	NA[b]	1 (1.1)		0	0	0	0 (0)
Lauren classification	Intestinal	59 (63.4)	19 (29.2)	82 (75.2)	5 (12.5)	139 (32.2)	150 (50)
	Diffusive	31 (33.3)	30 (46.2)	11 (10.1)	24 (60)	280 (64.8)	142 (47.3)
	Mixed	2 (2.2)	12 (18.5)	5 (4.6)	0	13 (3.0)	8 (2.7)
	NA[b]	1 (1.1)	4 (6.2)	11 (10.1)	11 (27.5)	0	0 (0)
Adjuvant chemotherapy	Yes	67 (72.0)	49 (75.4)	39 (35.8)	40 (100)[d]	432 (100)	144 (48)
	No	26 (28.0)	16 (24.6)	70 (64.2)	0	0	156 (52)
Radiation therapy	Yes	0	0	0	18	432	61 (20.3)
	No	93	65	109	22	0	239 (79.7)
No. of Recurrence		16	27	53	32[e]	177	125
Median follow-up (mo)		16.4	37.7	47	16.4	57.3	20.4

Abbreviations: AJCC, American Joint Committee on Cancer; KUCM, Kosin University College of Medicine; KUGH, Korea University Guro Hospital; MDACC, The University of Texas MD Anderson Cancer Center; SMC, Samsung Medical Center; YUSH, Yonsei University Severance Hospital

[a]Values represent number (percentage) of patients unless otherwise specified.

[b]Not available

[c]AJCC 6th edition and baseline stage is used for MDACC cohort

[d]Neoadjuvant chemotherapy

[e]Death

Supplementary Table 2. Upstream regulators of the mesenchymal phenotype signature.

Upstream Regulator	Predicted Activation State	Activation z-score	*P*-value	number of genes
TGFB1*	Activated	4.76	$5.9 X 10^{-20}$	65
NFκB	Activated	2.897	0.01	13
PRL	Activated	2.848	$4.4 X 10^{-6}$	12
MTPN	Activated	2.813	$4.3 X 10^{-6}$	8
WNT3A	Activated	2.783	$1.9 x 10^{-13}$	20
SP1	Activated	2.762	0.001	14
IL1B	Activated	2.69	$1.3 X 10^{-5}$	24
TGFB3*	Activated	2.646	$1.2 X 10^{-6}$	9
TP53	Activated	2.593	$6.7 x 10^{-8}$	38
SMAD3*	Activated	2.505	$3.9 10^{-8}$	14
NEUROG1	Inactivated	-2.309	$8.4 x 10^{-12}$	12
MGEA5	Inactivated	-2.309	$9.6 X 10^{-6}$	12
LRP1	Inactivated	-2.412	$2.3 X 10^{-7}$	6
SPDEF	Inactivated	-2.588	$1.3 X 10^{-5}$	7
MYC	Inactivated	-2.75	$2.2 X 10^{-6}$	27
CR1L	Inactivated	-2.985	$7.2 x 10^{-12}$	9
SMAD7*	Inactivated	-3.124	$1.6 X 10^{-6}$	10
MYCN	Inactivated	-3.13	$9.7 X 10^{-5}$	11
AHR	Inactivated	-3.246	$1.5 X 10^{-7}$	17
α-catenin	Inactivated	-4.058	$1.1 x 10^{-14}$	17

* Genes in TGF-beta pathway.

Supplementary Table 3. Univariate and multivariate analyses of clinical variables associated with recurrence free survival of patients with gastric adenocarcinoma in pooled cohorts (KUGH, YUSH, KUCM, SMC, and ACRG cohorts, n = 999).

	Univariate		Multivariate	
	Hazard ratio (95% CI)	*P* value	Hazard ratio (95% CI)	*P* value
Sex (M or F)	1.02 (0.83-1.24)	0.84	1.1 (0.89-1.35)	0.37
Age (>65 years)	1.38 (1.11-1.71)	0.003	1.32 (1.06-1.65)	0.01
Tumor location (upper or middle/lower)	1.1 (0.85-1.45)	0.42	1.08 (0.82-1.4)	0.56
Depth of tumor invasion (T1/T2 or T3/T4)	2.5 (2.1-3.0)	2×10^{-16}	1.93 (1.57-2.37)	$3.69 \text{ x } 10^{-10}$
Lymph node invasion (presence or absence)	2.1 (1.5-2.8)	$7.5 \text{ x } 10^{-6}$	1.86 (1.34-2.6)	0.0002
Distant metastasis (presence or absence)	4.6 (3.2-6.6)	2.2×10^{-16}	3.0 (2.05-4.4)	$1.4 \text{ x } 10^{-8}$
Lauren classification (diffuse/mixed or intestinal)	1.15 (0.95-1.4)	0.1	1.14 (0.93-1.4)	0.19
AJCC stage (I/II or III/IV)	2.6 (2.0-3.4)	4.9×10^{-12}	1.8 (1.34-2.41)	$8.5 \text{ x } 10^{-5}$
MP signature (MP or EP)	2.34 (1.9-2.9)	1.2×10^{-15}	1.75 (1.4-2.2)	$5.31 \text{ x } 10^{-7}$

Abbreviations: AJCC, American Joint Committee on Cancer; CI, confidence interval; KUGH, Korea University Guro Hospital, YUSH, Yonsei University Severance Hospital, KUCM, Kosin University College of Medicine; SMC, Samsung Medical Center; ACRG, Asian Cancer Research Group).

Supplementary Table 4. Association of prognostic subtypes with Lauren histologic type in five cohorts (KUGH, YUSH, KUCM, SMC, TCGA, and ACRG cohort, n = 1261[a]).

**Characteristics**		**MP**	**EP**	**Total No.**
No. of patients		252	915	1167
Lauren Subtypes	Intestinal	96 (38.1%)	519 (56.7%)	615
	Diffuse	156 (61.9%)	396 (43.3%)	552

[a] Of 1261 tumor samples, 94 were not included in the analysis owing to lack of information about Lauren histologic type (n = 27) or mixed histologic features (n = 67).
$P = 1.5 \times 10^{-7}$ by χ^2-test

Supplementary Table 5. Association of MP subtype with 4 TCGA molecular subtypes in TCGA cohort.

Characteristics		MP	EP	Total No.
No. of patients		70	192	262
TCGA Subtypes	CIN	22 (31.4%)	105 (54.7%)	127
	EBV	4 (5.7%)	20 (10.4%)	24
	GS	38 (54.3%)	16 (8.3%)	54
	MSI	6 (8.5%)	51 (3.5%)	57

CIN, Chromosomal instability
EBV, Epstein-Bar virus
GS, Genomically Stable
MSI, Microsatellite Instability

Supplementary Table 6. Primers used in pyrosequencing

Gene		Sequence	Annealing (℃) / number of cycles
IGF1	F	5'-biotin-GATGGGAGATGTTGAGAGTAATGTTA-3'	59 / 35
	R	5'-AAAAAAATCCTTACTCAATAACT-3'	
	S	5'-AAAACAAATAAAACCTAC-3'	
