# Supplementary material for: Clinical and genomic landscape of gastric cancer with a mesenchymal phenotype
Source: Nat Commun. 2018 May 3;9:1777. doi: 10.1038/s41467-018-04179-8 (PMC5934392; doi:10.1038/s41467-018-04179-8)
Supplement: Supplementary file 3 — Description of Additional Supplementary Files [file 41467_2018_4179_MOESM3_ESM.pdf]

Description of Additional Supplementary Files

File Name: Supplementary Data 1
Description: Gene list of prognostic signature.

File Name: Supplementary Data 2
Description: Clinical data of each cohort.
